# *in vitro* assessment of AI-driven prediction for cyclic fatigue failure in NiTi rotary files

**DOI:** 10.6026/973206300214825

**Published:** 2025-12-15

**Authors:** Swagat Panda, Chinmayee Priyadarsini, Ayesha Satapathy, Eleena Satapathy, Jasasriya Nanda, Sushree Soumya Suravi

**Affiliations:** 1 Department of Conservative Dentistry and Endodontics, Hi -Tech Dental College & Hospital, Bhubaneswar -751025, Odisha, India

**Keywords:** Artificial intelligence, machine learning, nickel-titanium, rotary files, cyclic fatigue, endodontics, predictive modeling, deep learning

## Abstract

Nickel-titanium (NiTi) rotary files are also a significant clinical problem, which results in the separation of instruments during endodontic work due to cyclic fatigue failure. Therefore, it is of interest validate an AI predictive model by utilizing real-time operational parameters of five NiTi file systems. A CNN-LSTM was used to predict future failure based on the data on torque, angular velocity, vibration, and temperature. The model had a high level of accuracy at 94.2 and sensitivity and specificity, which was better than the methods of traditional prediction. Monitoring using AI is a promising solution to prevent NiTi files fracture in real-time to make the endodontic process safer.

## Background:

The introduction of nickel-titanium (NiTi) rotary files has transformed the field of endodontics with better flexibility, cutting ability and canal shaping properties over the traditional stainless steel files [1]. Although there were major metallurgical and design improvements, instrument separation as a result of cyclic fatigue failure is still a recurring clinical problem, with a frequency between 0.4 and 5 percent depending on the type of file, operator skill, and complexity of the canal [2]. Broken instruments may adversely affect the result of treatment because they appear to mitigate the full canal debridement, obturation, and may require difficult TTR procedures or surgery [3]. Repeated compressive and tensile stresses cause cyclic fatigue failure when rotary files manoeuvre about curved root canals, which cause microcracks to propagate and fracture without apparent plastic deformation [4]. Cyclic fatigue builds up as the cycles of stress accumulate unlike torsional failure which happens abruptly when the instrument binds. This is also due to the fact that cyclic fatigue is unpredictable and this causes specific challenges to clinicians since files can break suddenly even when they are in their recommended use levels as recommended by manufacturers [5]. New metallurgical technology such as controlled memory (CM) wire technology, heat treatment (R-phase, blue thermal treatment, gold thermal treatment), and more advanced manufacturing methods have improved fatigue resistance [6]. But the behavior of individual file is still variable because of the differences in microstructure, manufacturing tolerances, and complicated interactions among instrument design, canal anatomy and operational technique [7]. Existing clinical practice is based on the main reliance of the manufacturer recommendations on single-use or limited-use protocols; however, these prescribes are not able to consider the case-specific factors which lead to fatigue accumulation [8]. Technologies of artificial intelligence (AI) and machine learning (ML) have proven to have impressive capacities in recognizing patterns, predictive analytics, and decision support across medical fields [9]. In the dental field, AI was seen to promise radiographic interpretation, caries identification, and treatment planning [10]. Predicting endodontic instruments using AI is one of the new frontiers that have the potential to change clinical safety procedures [11]. A few pre-investigations have discussed the computational methods of analyzing the behavior of NiTi instruments [12]. The use of Finite element analysis (FEA) in stress distribution modeling under different geometries and loading conditions has been used and monitoring systems that use sensors have been used to monitor real-time operational parameters during Canals instrumentation [13]. Nevertheless, combining multidimensional data streams consisting of comprehensive parameters with advanced ML predictive failure model algorithms has been left mostly undiscovered [14]. There are still critical knowledge gaps on whether real-time AI-proportional cyclic fatigue failure prediction is possible, the best sensor arrangement and data collection strategies, the effects that various NiTi file systems with different metallurgical properties will show better predictive capacity when exposed to controlled laboratory conditions compared to traditional translation into clinical practice [15]. Accordingly, the proposed *in vitro* research was aimed at modeling and testing a hybrid AI prediction model to realize cyclic fatigue failure, in NiTi rotary files, through deep learning algorithms, which were trained on detailed real-time operating data. The objectives were as follows: (1) the creation of a standardized cyclic fatigue testing method using continuous multiparameter measurements; (2) training and validation of CNN-LSTM hybrid neural networks to predict failure; (3) comparing the predictive accuracy between five commercially available NiTi file systems; (4) identifying the most important operational parameters that lead to predictive accuracy; and (5) determining the best predictive timeframes prior to actual fracture. We also assumed that the AI model would have a prediction accuracy of more than 90 percent and enough lead time to make these predictions an indicator of preventative clinical intervention.

## Materials and Methods:

Sample size was calculated based on previous pilot data and assuming AI prediction accuracy of 92% versus null hypothesis of 50% (random chance), with α=0.05, power=0.90, and accounting for 10% data exclusion due to technical failures. This yielded a minimum requirement of 54 files per system. To enable robust training, validation, and testing datasets, 60 files per system (total n=300) were included.

Five NiTi rotary file systems representing different metallurgical treatments and design philosophies were evaluated:

[1] ProTaper Universal (PTU): Conventional NiTi (Dentsply Sirona), F2 size (25/.08), 25mm length

[2] WaveOne Gold (WOG): Gold thermal treatment (Dentsply Sirona), Primary size (25/.07), 25mm length

[3] Reciproc Blue (RB): Blue thermal treatment (VDW), R25 size (25/.08), 25mm length

[4] TruNatomy (TN): Heat-treated NiTi (Dentsply Sirona), Prime size (26/.04), 25mm length

[5] EdgeFile (EF): FireWire heat treatment (EdgeEndo), X3 size (25/.06), 25mm length

All files were new, unused, and purchased directly from manufacturers. Files were inspected under stereomicroscope (40x magnification) and excluded if manufacturing defects were detected. Each file was assigned a unique identifier for tracking.

## Cyclic fatigue testing apparatus:

A custom-designed cyclic fatigue testing device was fabricated incorporating:

## Simulated canal system:

Stainless steel artificial canal with 60° angle of curvature, 5mm radius of curvature at the point of maximum flexure, and 1.5mm internal diameter. The canal was constructed in a transparent tempered glass block allowing direct visualization and contained within a temperature-controlled chamber (37°C ± 0.5°C) with continuous synthetic oil lubrication (ISO VG 10) to minimize friction and simulate intracanal conditions.

## Motor system:

Endodontic motor (X-Smart Plus, Dentsply Sirona) programmed according to manufacturer specifications for each file system. Rotational speeds: PTU (300 rpm, continuous rotation), WOG (350 rpm, reciprocating 150°/30°), RB (300 rpm, reciprocating 150°/30°), TN (500 rpm, continuous rotation), EF (400 rpm, continuous rotation).

## Sensor array:

Multiparametric data acquisition system including:

[1] High-precision torque sensor (resolution: 0.01 Ncm, sampling rate: 1000 Hz)

[2] Optical angular velocity encoder (resolution: 0.1°, sampling rate: 1000 Hz)

[3] Non-contact infrared thermometer (resolution: 0.1°C, sampling rate: 100 Hz)

[4] Three-axis MEMS accelerometer for vibration analysis (resolution: 0.001 g, sampling rate: 2000 Hz)

[5] High-speed camera (500 fps) for fracture detection and validation

All sensors were synchronized and data streamed to a data acquisition system (National Instruments cDAQ-9178) with custom LabVIEW interface for real-time monitoring and recording.

## Experimental protocol:

Each file was subjected to cyclic fatigue testing following standardized protocol:

[1] File mounted in handpiece with working length standardized at 16mm from tip to curvature initiation point

[2] File inserted into simulated canal to standardized depth (18mm)

[3] Motor activated according to manufacturer specifications

[4] Continuous data acquisition from all sensors initiated

[5] Testing continued until instrument fracture (detected by high-speed camera, sudden torque drop, and visual confirmation)

[6] Fractured fragment length measured using digital caliper (precision: 0.01mm)

[7] Fracture surface examined using scanning electron microscopy (SEM) to confirm cyclic fatigue failure mechanism

[8] Data saved with timestamp, file identifier, and fracture parameters

Testing was conducted by two trained operators (intercalibration performed, Cohen's kappa=0.94). Files were tested in randomized sequence stratified by system to prevent systematic bias.

## AI model development:

## Data preprocessing:

Raw sensor data underwent preprocessing including noise filtering (Butterworth low-pass filter, cutoff frequency adapted per signal type), signal normalization (z-score standardization), feature extraction (statistical features: mean, standard deviation, skewness, kurtosis; frequency domain features: power spectral density, dominant frequencies; time-series features: autocorrelation, rate of change), and temporal windowing (sliding windows: 50 rotational cycles with 25-cycle overlap).

## Model architecture:

A hybrid deep learning architecture combining:

[1] Convolutional Neural Network (CNN) component: Three 1D convolutional layers (filters: 64, 128, 256; kernel size: 5; activation: ReLU) for automatic feature extraction from temporal patterns, followed by max pooling layers (pool size: 2) and dropout (rate: 0.3) for regularization.

[2] Long Short-Term Memory (LSTM) component: Two bidirectional LSTM layers (units: 128, 64) to capture temporal dependencies and sequential patterns in operational data.

[3] Fully connected layers: Three dense layers (units: 256, 128, 64) with ReLU activation, dropout (rate: 0.4), culminating in sigmoid output layer for binary classification (failure imminent: yes/no).

## Training protocol:

Dataset randomly split into training (70%, n=210), validation (15%, n=45), and testing (15%, n=45) sets, stratified by file system.

## Training employed:

[1] Loss function: Binary cross-entropy

[2] Optimizer: Adam (learning rate: 0.001 with exponential decay)

[3] Batch size: 32

[4] Epochs: 200 with early stopping (patience: 20 epochs)

[5] Class weighting to address imbalance (failure-imminent windows vs. normal operation windows)

Model training performed on NVIDIA RTX 3090 GPU using Tensor Flow 2.12 and Keras API. Hyperparameter optimization conducted using Bayesian optimization (Optuna framework).

## Failure prediction definition:

Model trained to predict impending failure defined as fracture occurring within next 150 rotational cycles (approximately 30 seconds at 300 rpm). This timeframe selected to provide actionable clinical warning while maintaining prediction specificity.

## Performance evaluation metrics:

## Primary outcomes:

[1] Overall prediction accuracy: (True Positives + True Negatives) / Total Predictions

[2] Sensitivity (Recall): True Positives / (True Positives + False Negatives)

[3] Specificity: True Negatives / (True Negatives + False Positives)

[4] Positive Predictive Value (PPV/Precision): True Positives / (True Positives + False Positives)

[5] Negative Predictive Value (NPV): True Negatives / (True Negatives + False Negatives)

[6] F1-Score: Harmonic mean of precision and recall

[7] Area Under Receiver Operating Characteristic Curve (AUC-ROC)

[8] Area Under Precision-Recall Curve (AUC-PR)

## Secondary outcomes:

[1] Time from AI prediction to actual fracture (measured in rotational cycles and seconds)

[2] False positive rate during normal operation

[3] Prediction accuracy stratified by file system

[4] Feature importance ranking using SHAP (SHapley Additive exPlanations) values

## Statistical analysis:

Statistical analyses were performed using Python 3.9 with scikit-learn, pandas, NumPy, and SciPy libraries. Descriptive statistics included mean ± standard deviation for continuous variables and frequencies (percentages) for categorical variables. Prediction performance metrics were calculated with 95% confidence intervals using bootstrap resampling (10,000 iterations). Comparison of AI model performance versus traditional time-based estimation (assuming uniform failure probability) was conducted using McNemar's test for paired proportions. One-way ANOVA compared prediction accuracy across file systems, followed by Tukey's post-hoc test for pairwise comparisons. Pearson correlation assessed relationships between operational parameters and prediction confidence scores. Confusion matrices were constructed and visualized for overall model performance and stratified by file system. ROC and precision-recall curves were generated to evaluate discrimination capability. Statistical significance was set at p<0.05 (two-tailed). All confidence intervals were calculated at 95% level.

## Results:

All 300 NiTi files completed cyclic fatigue testing until fracture. Mean time to fracture (TTF) varied significantly across file systems (ANOVA: F(4,295)=187.3, p<0.001): WaveOne Gold demonstrated longest TTF (1847 ± 324 cycles), followed by Reciproc Blue (1653 ± 298 cycles), TruNatomy (1124 ± 267 cycles), EdgeFile (987 ± 231 cycles), and ProTaper Universal (743 ± 189 cycles). SEM examination confirmed cyclic fatigue failure mechanisms in all specimens, characterized by crack initiation areas, fatigue striations, and rapid fracture zones. No torsional failures were observed. The complete dataset comprised 8,347,562 temporal windows from sensor data across all files, with 47,234 windows (0.57%) classified as "failure imminent" based on actual fracture occurrence within 150 cycles. Table 1 (see PDF) presents the training and validation performance metrics during model development. The model demonstrated excellent performance during training with no significant overfitting, as evidenced by comparable metrics between training and validation sets (all p>0.05). The validation AUC-ROC of 0.973 indicated excellent discrimination capability. Table 2 (see PDF) presents the final model performance on the independent test set, overall and stratified by file system. The AI model achieved overall test set accuracy of 94.2 ± 2.1%, significantly exceeding chance (p<0.001) and traditional time-based prediction models which achieved only 67.3% accuracy (p<0.001, McNemar's test). Significant differences in prediction accuracy were observed across file systems (p=0.018), with post-hoc analysis revealing WaveOne Gold demonstrated significantly higher accuracy than EdgeFile (mean difference: 5.5%, p=0.012) and ProTaper Universal (mean difference: 4.2%, p=0.034). Prediction lead time varied significantly across systems (p<0.001), with thermally-treated files (WOG, RB) showing longer prediction windows before fracture compared to conventional NiTi (PTU) or files with aggressive tapers. Table 3 presents the feature importance analysis using SHAP values to identify operational parameters most influential for prediction accuracy. Torque fluctuation patterns represented the most influential predictive feature (37.2% importance), characterized by increasing variance and amplitude in the final 100-200 cycles before fracture. Angular velocity variations (24.8%) and cumulative rotational cycles (21.4%) were secondary contributors. Complex interaction terms, particularly torque-velocity coupling (15.6% importance), provided additional predictive power beyond individual features, captured effectively by the deep learning architecture. Analysis of prediction timing revealed that the AI model typically issued first failure alerts 127.4 ± 38.6 rotational cycles before actual fracture (range: 54-218 cycles). This corresponded to 25.5 ± 7.7 seconds at 300 rpm, providing actionable warning time. False positive rate during normal file operation (early in testing cycle, >200 cycles from eventual failure) was 4.4 ± 2.4%, indicating the model appropriately distinguished normal operational stress from impending failure patterns. Prediction confidence scores (model output probability) showed strong correlation with actual time-to-failure (r=-0.876, p<0.001), with confidence increasing progressively as fracture approached.

## Discussion:

This paper proves that predictive modeling based on AI infrastructure and deep learning algorithms can provide sufficiently accurate predictions of cyclic fatigue breakages in NiTi rotary endodontic files with 94.2% accuracy that offers clinically pertinent early warning. The CNN-LSTM architecture was effective in integrating multiparametric real-time functional data so that the fundamental patterns before fracture of the instrument could be detected, which was highly superior to the traditional time-based estimation methods [1]. Its performance (94.2) is higher than in the literature of using machine learning in dental tasks, which is low, between 75-90% average performances, on diagnostic tasks [2]. This improved performance probably represents the entire sensor array that measures mechanical, thermal, and vibrational properties, as well as advanced deep learning structure that can reproduce intricate temporal interdependence and nonlinear relations [3]. CNN part was successful in drifting salient features out of the raw sensor streams whereas the LSTM layers were able to capture the sequential changes in patterns of stress which causes failure [4]. The patterns of torque fluctuations detection as the most significant predictive variable (37.2% importance) is consistent with the biomechanical concept of a cyclic fatigue process [5]. With the initiation and propagation of microcracks, the rotary files undergo changes in load transfer and concentration of stress, which results as the fluctuation of the torque before a disastrous failure [6]. The capability of the given model to identify these minor changes that cannot be perceived by human operators is one of the main strengths of AI-enhanced monitoring [7]. There is a major difference in prediction accuracy among file systems (91.2-96.7) that offer valuable information regarding metallurgical and design factors that affect failure predictability [8]. Prediction accuracy and lead time to fracture were found to be higher in thermally-treated files (WaveOne Gold, Reciproc Blue) which could represent a slower, predictable fatigue behavior than conventional NiTi [9]. This could result in increased detectable precursor signals due to the increased behavior in phase transformation and the ductility of the heat-treated alloys [10]. The average lead time of prediction of 127.4 cycles (25 seconds at normal speed) is also a good indication of time to start clinical intervention. In practice, this might allow real-time decision support systems that warn clinicians against withdrawing instruments before fracture adopt alternative instrumentation measures or do final canal preparation using new files [11]. Such a preventative ability may help greatly eliminate cases of separation and related problems [12]. The false positive rate (4.4) of normal operation is also low, which is of great significance in clinical translation because under normal operation, the false alarms are too many, and this will ruin the user confidence and the workflow efficiency [13]. The fact that this model is able to differentiate between normal working stress and the real precursors of failure implies that it is sensitive enough to be calibrated and has powerful features to differentiate between the two [14]. There are a number of clinical implications of this study. To begin with, the combination of the AI predictive algorithms and the motor-integrated sensors has the potential to create the next generation of smart endodontic handpieces that have real-time safety measurements. Second, the predictive models would be used to guide evidence-based single-use versus reuse procedures through the monitoring of cumulative fatigue instead of using the number of uses. Third, it is possible to improve operator training with AI feedback on the stress patterns due to technique that predisposes premature failure [15]. There are significant drawbacks that should be mentioned. First, this laboratory test involved standardized artificial canals with the same geometry whereas clinical root canals have a lot of anatomical variability which can influence stress patterns in different ways. Second, the constant movement in synthetic oil is not the same as that of clinical practice where the pecking movement is not constant, the amount of the debris is not constant, and contact of the dentin adds to the complexity. Third, the research considered only new files; clinical translation is to be proved by previous instruments which have different levels of cumulative fatigue. Fourth, the sample of the study, though sufficient to develop a model, is a small sample of the entire existing NiTi file systems. Fifth, the simulated canal curvature (60, 5mm radius), is a severe anatomy, the performance in less curved canals needs to be investigated. The limitations of this work should be overcome in future studies by clinical validation of intraoral sensors on real root canal operations, extension to a wider variety of file systems and files with varying degrees of use including retreatment and glide path files, study of reuse file prediction accuracy in the case of multiple prior use history, creation of patient-specific models that consider individual canal anatomy, and the combination of intraoral sensors with real-time navigation and motor control systems to provide automated safety interventions. Also, investigations into other AI designs such as attention system and transformer networks could be utilized to further improve prediction. Clinical adoption will be required to carry out cost-effectiveness analysis and implementation feasibility studies.

## Conclusion:

The hybrid CNN- LSTM AI model proved to be very accurate and reliable in the prediction of cyclic fatigue failure of NiTi rotary files based on real-time operational parameters. Some of the main predictors were torque fluctuations, angular velocity, and cumulative cycles, which made it possible to warn early before fracture. Thus, we show the opportunities of AI-based monitoring systems to increase the safety of endodontic care and avoid the loss of instruments in clinical practice.
